# Developing the Effective Method of Spectral Harmonic Energy Ratio to Analyze the Arterial Pulse Spectrum

**DOI:** 10.1093/ecam/neq054

**Published:** 2011-01-11

**Authors:** Chin-Ming Huang, Ching-Chuan Wei, Yin-Tzu Liao, Hsien-Cheh Chang, Shung-Te Kao, Tsai-Chung Li

**Affiliations:** ^1^School of Chinese Medicine, China Medical University, Taichung County, Taiwan; ^2^Department of Information and Communication Engineering, Chaoyang University of Technology, Taichung, Taiwan; ^3^Graduate Institute of Biostatistics, China Medical University, Taichung County, Taiwan

## Abstract

In this article, we analyze the arterial pulse in the spectral domain. A parameter, the spectral harmonic energy ratio (SHER), is developed to assess the features of the overly decreased spectral energy in the fourth to sixth harmonic for palpitation patients. Compared with normal subjects, the statistical results reveal that the mean value of SHER in the patient group (57.7 ± 27.9) is significantly higher than that of the normal group (39.7 ± 20.9) (*P*-value = .0066 < .01). This means that the total energy in the fourth to sixth harmonic of palpitation patients is significantly less than it is in normal subjects. In other words, the spectral distribution of the arterial pulse gradually decreases for normal subjects while it decreases abruptly in higher-order harmonics (the fourth, fifth and sixth harmonics) for palpitation patients. Hence, SHER is an effective method to distinguish the two groups in the spectral domain. Also, we can thus know that a “gradual decrease” might mean a “balanced” state, whereas an “abrupt decrease” might mean an “unbalanced” state in blood circulation and pulse diagnosis. By SHER, we can determine the ratio of energy distribution in different harmonic bands, and this method gives us a novel viewpoint from which to comprehend and quantify the spectral harmonic distribution of circulation information conveyed by the arterial pulse. These concepts can be further applied to improve the clinical diagnosis not only in Western medicine but also in traditional Chinese medicine (TCM).

## 1. Introduction

**Figure 1:**
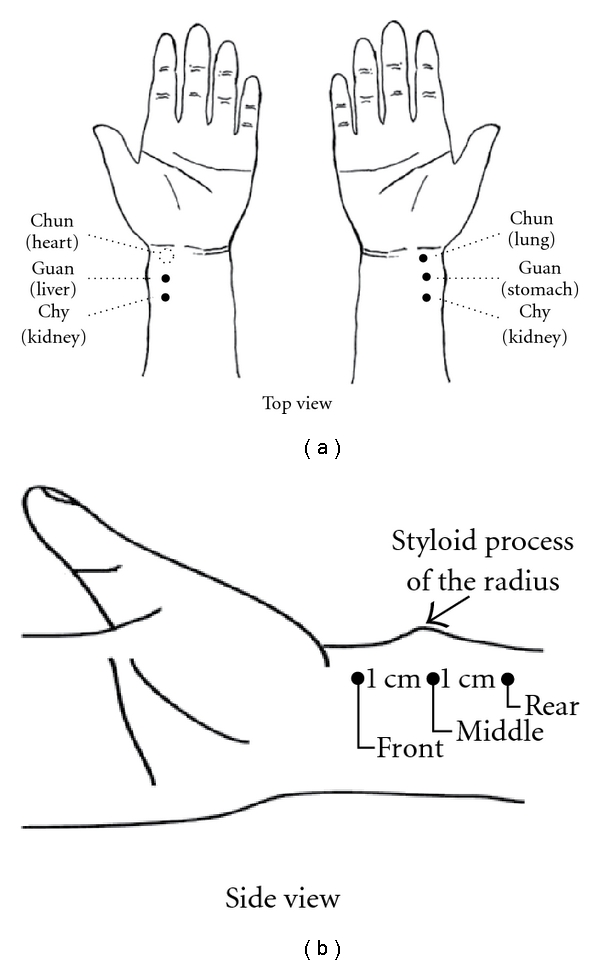
(a) The locations of “Chun", “Guan" and “Chy" as well as the related organs are demonstrated. The measurement position of the radial arterial pulse is at the “Chun" site (dashed-line circle) on the wrist of the left hand. (b) The side view of the locations of Chun, Guan and Chy.

**Figure 2:**
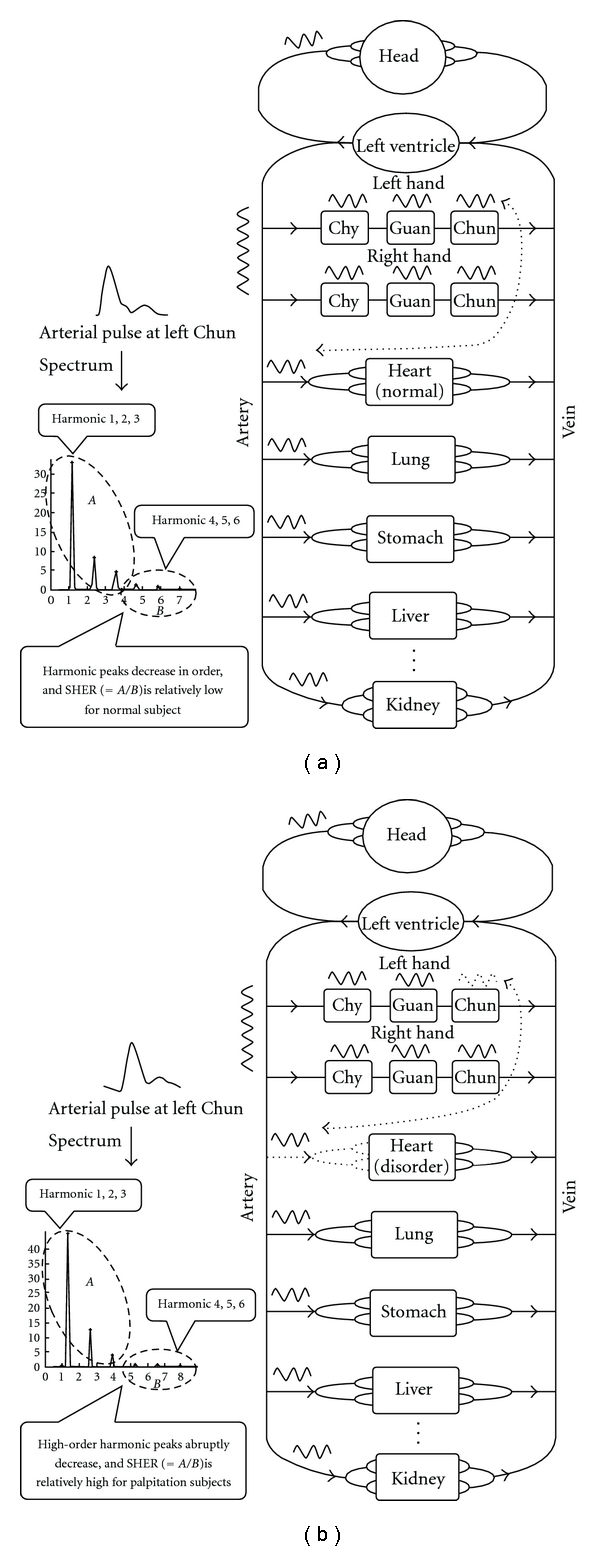
(a) The arterial pressure wave pumped by left ventricle flows into both organs and hands. Thus, the blood circulation forms a wave system. In the arterial pulse spectrum of a normal subject, the harmonic peaks decrease progressively. Hence, SHER is relatively low. (b) For a subject with heart disorder, the arterial pressure wave flowing into the organs might be influenced and the harmonic peaks decrease abruptly in the fourth, fifth and sixth harmonics. Thus, there is less energy in the above harmonic frequencies for the patient subject and SHER is relatively high.

The time-domain arterial pulse contour detected by a non-invasive method is essentially a significant signal to aid in comprehending the cardiovascular system in Western medicine [1, 2]. Most researches on the arterial pulse in Western medicine have focused on the application of the related parameters, such as compliance and pulse wave velocity (PWV), to assess the elasticity and stiffness of the artery [3–8]. Therefore, in the clinical setting, these parameters become useful indexes of hypertension or cardiovascular risk factors. In the same way, the arterial pulse conveys much information about the condition of Qi, blood and organs (Zang-Fu) derived from the pulse diagnosis theory in TCM. For nearly 2000 years, pulse examination has served as a general guide for clinical diagnosis in TCM. It is obtained when the physician places the tips of the index, middle and fourth fingers on the wrist above the radial artery of the patient's left or right hand. For the physicians of TCM, pulse diagnosis involves feeling the variation or pulsation of arterial pulse waveform in the time domain with the fingertips; thus, normal and abnormal pulse patterns could be identified to diagnose the status of the human body. In TCM, the relationships between the pulse-taking positions and the organs are demonstrated in Figures 1(a) and 1(b). For example, the pulse at the right “Chun” position is related to the lungs, the pulse at the right “Guan" position is connected with the stomach, and the pulse at the left “Chun" portion is associated with the heart. Although these relationships have not been scientifically verified, the fact that the pulses detected at “Chun", “Guan" and “Chy" signify different clinical meanings has been confirmed [9]. Furthermore, from the viewpoint of wave transmission, the arterial pressure wave pumped by the left ventricle is transmitted not only to the organs but also to the hands, as shown in Figure 2(a). Because the human body forms a closed and linked wave system, the fluctuation of the pressure wave into organs resulting from disease might induce a corresponding variation of pressure wave in the hands according to the wave transmission theory [10, 11]. Therefore, it is reasonable to believe that the status of organs can be determined by the arterial pressure pulse found in the hands.

Because arterial pulse-related information is hard to find in the time domain by the fingertips, the analysis in the spectral domain provides further insight into the relation between disease and the arterial pulse. As for spectral domain analysis, the theory of organ resonance was proposed. This theory states that the heart will resonate with organs to reduce the resistance of the arterial pulse propagating into the organs [12–15]. With regard to this theory, the resonance frequencies of organs correspond to the spectral-domain harmonics of the arterial pulse, respectively. For example, the spleen is related to the 3rd harmonic and the stomach to the fifth harmonic, and so forth. Furthermore, the cardiovascular system is an electrically driven mechanical-pumping system, and the organ resonance theory is verified via the accordance of the spectral distribution of the electrocardiogram (ECG) as well as the arterial pulse [16, 17]. Furthermore, the specifically different patterns of the harmonic components of the arterial pulse among surviving and expired rats that suffered from acute bleeding and progressive hemorrhaging were studied [18, 19]. However, the previously mentioned spectral-domain studies focus on the frequency range of 0–10 Hz, where most arterial pulse energy is concentrated.

As for the spectral energy of the arterial pulse above 10 Hz, the spectral energy ratio at frequency *f* (SER(*f*)) was defined as: SER(*f*) = *E*_1_(*f*)/*E*_2_(*f*), and proposed as a parameter to assess the balance of energy. *E*_1_(*f*) is the integrated spectral energy below *f* Hz, and *E*_2_(*f*) is that above *f* Hz [20]. It was found that the arterial pulse spectrum above 10 Hz fell sharply for normal subjects, but it had large variations beyond 10 Hz for patients. Furthermore, the spectral energy would increase above 10 Hz when internal organs were damaged or functional disorder existed. Therefore, according to their research, SER(10) = 100 appears as an index above which health is normal and below which health is abnormal. Aside from these researches, few studies pertain to the spectral analysis of the arterial pulse.

In clinical observation of the harmonic distribution of the arterial pulse spectrum below 10 Hz, we found that the spectral harmonics of the arterial pulse gradually decayed in sequence from low- to high-order harmonics for most of the normal subjects; however, for cardiovascular patients, the harmonics above the fourth always over decayed. The schematic diagrams of the spectrum of this phenomenon are illustrated in Figure 2(b). Therefore, this interesting phenomenon inspired us to develop the spectral analysis method, SHER, to quantify such an effect. We collected the pulses of patients with the symptom of palpitation as well as the pulses of normal subjects, and we analyzed them with SHER. Finally, a statistical method was used to determine the differences of SHER between the patient group and the normal group, and then to validate this phenomenon.

## 2. Methods

### 2.1. Normal and Palpitation Subjects

Thirty normal subjects (N group) who had no reported cardiovascular diseases (average age of 40.2 ± 8.3) were chosen by the doctor to take the radial arterial pulse from February 15, 2009 to September 15, 2009. The arterial pulse of 30 palpitation patients (P group) (average age of 41.9 ± 10.4 years) was also measured. There is no significant difference in age between N and P groups because *P*-value = (−.2699) is >.05. All subjects were asked not to have any alcoholic or caffeinated beverages on the day of the experiment, and all subjects rested for 20 min prior to pulse measurement. The protocol and informed consent were approved and the written informed consents were obtained from all participants before they enrolled in this study. The reason for selecting patients with cardiovascular-related diseases such as palpitation is that we considered such subjects apt to reveal cardiovascular instability. None of the authors in this study had any conflicts of interest.

Palpitation is characterized by a very fast series of heartbeats, skipped beats or thumping in the chest, from lasting more than one, to several minutes. These patients were diagnosed through medical histories, physical examinations and resting ECG. The inclusion criterion is the palpitation patients with non-arrhythmic cardiac problems, and they are not associated with potentially more serious symptoms of dizziness, or syncope. We excluded the subjects with palpitation caused by thyroid disease, overexertion, drugs, food (caffeine, tea), anxiety or panic, and those whose ECG showed specific abnormality.

### 2.2. Pulse Measurement Experiment

**Figure 3:**
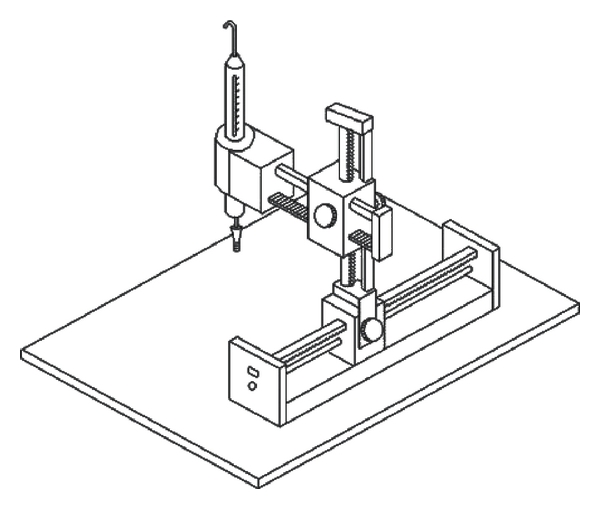
The schematic diagram of the pulse-acquiring instrument is illustrated.

The schematic diagram of the pulse-acquiring instrument is illustrated in Figure 3. The locations of “Chun", “Guan" and “Chy" are demonstrated in Figure 1(a), and the measurement position of the radial arterial pulse is at the “Chun" site on the wrist of the left hand, where it is adjacent to the ventral portion of the styloid process of the radius on the distal side. According to TCM, this measurement location is the so-called left “Chun", which is related to the function of the heart [21]. The initial medical book of Chinese medicine, the “Huang-Ti-Nei-Ching", presented such a relationship [22]. However, there is no related research on this viewpoint. The left radial artery is closer to the heart than the right artery. Furthermore, the radial pressure pulse, which propagates from the heart to the end of the radial artery, consists of forward and backward travelling waves. The “Chun" position is closer to the palm, which causes fewer reflection waves due to more artery branches than is the case with the “Guan" and “Chy" positions. So, the arterial pulse at the left “Chun" position is less influenced than other positions by reflection waves. As a result, it might reflect the cardiac function. When it is not regular, the heart may have some problems. This is the reason for choosing the arterial pulse at this site to investigate palpitation patients. In the experiments, the N and P groups accepted pulse measurements separately, and each subject was seated while undergoing examination. All subjects were asked not to have any alcoholic or caffeinated beverages on the day of the experiment and rested for 20 min prior to pulse measurement. 

A self-designed pulse recorder was used to record the radial arterial pulse with a strain-gauge pressure sensor with high linearity. The sensitivity, non-linearity and resistance of the sensor are about 0.9 mV/V, ±1% rated output and 350 *Ω*, respectively. The frequency response of the system is about 0-1 kHz. The pulse value is approximately calibrated by both the height of the maximum arterial pulse peak and the related systolic blood pressure measured with the cuff sphygmomanometer. The criterion of a suitable pressure is meant to facilitate seeking the largest pulse amplitude. The pressure sensor is mounted in a holder that allows it to travel in three-dimensional directions. The wrist has to be held motionless and adjusted in such a way that the sensor is in direct contact with the skin at the desired position of the radial artery. All of the arterial pulses were measured by the same doctor and the same device. While the sensor has not touched the hand, the output signal from the sensor followed by a low noise amplifier (gain = 100) is recorded for about 10 s. It is defined as the background noise, which should be excluded by subtraction in the detected arterial pulse. This background noise signal is transformed into its power spectral density using a periodogram based on Fast Fourier Transform (FFT). The average power spectral density of the background noise in 0–10 Hz is calculated as about: 1.5 × 10^−15^ V^2^/Hz. It is far less than the power of the first six harmonics (≈10^−12^ V^2^/Hz) of the arterial pulse and thus has no influence on the calculation accuracy of SHER (Supplementary data). The electrical pulse from the sensor is digitized with the sampling rate of 4000 Hz. The pulse sequence of 10 s (about 10 pulses) is selected and fed into a computer for processing through FFT with a rectangular window to obtain the power spectral density.

### 2.3. Methods of Spectral Harmonic Energy Ratio

The pulse sequence with 10 s (about 10 pulses) is selected and fed into a computer for processing through Fast Fourier Transform with a rectangular window to obtain the power spectral density. Due to the overly decayed effect above the fourth harmonic in the arterial pulse spectrum, the harmonic energy above the fourth abruptly decreased. To characterize this effect, the parameter SHER modified from SER(*f*) is developed to evaluate this feature. Because the harmonic energy above the sixth harmonic is relatively small, it was neglected in the computation of SHER. Thus, SHER, which denotes the energy ratio between the total energy of harmonic 1–3 and 4–6, was defined as the following:

(1) $$\text{SHER=}\frac{\sum_{i=1}^{3}P_{i}}{\sum_{i=1}^{6}P_{i}}$$

*P*_*i*_ is *i*th harmonic peak value representing the harmonic energy is obtained by a peak detection program. The results were analyzed by Student's *t*-test to identify the significant distinction of the parameter SHER between the two groups of normal and patient subjects.

## 3. Results

**Figure 4:**
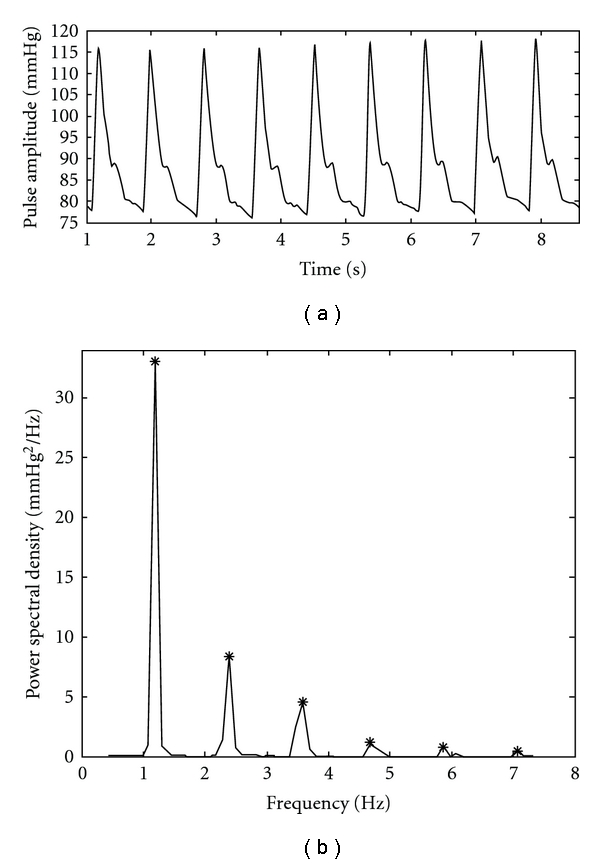
(a) The measured arterial pulse of a typical normal subject in the N group is shown. (b) The corresponding power spectral density is plotted and the star denotes the harmonic peak.

**Figure 5:**
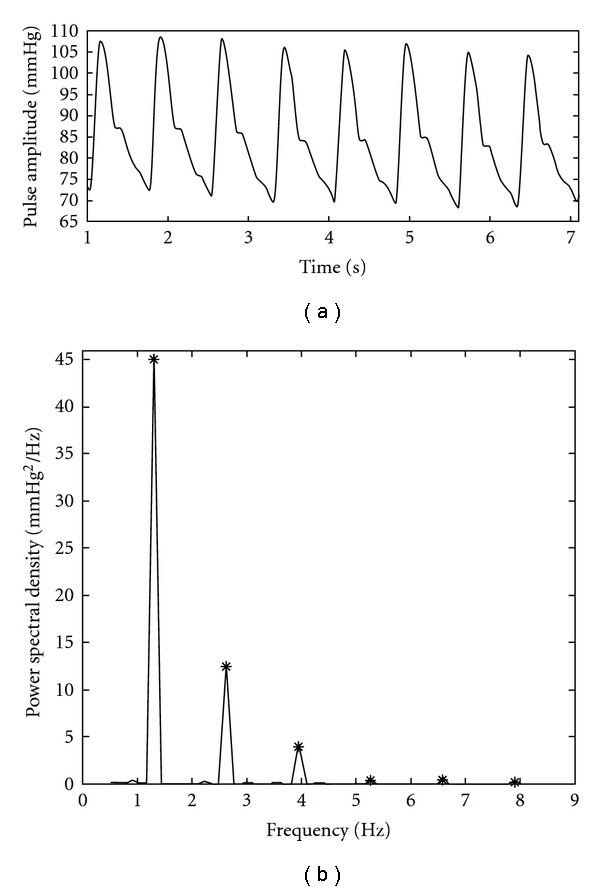
(a) The measured arterial pulse of a typical patient with palpitation in the P group is shown. (b) The related power spectral density is plotted and the star denotes the harmonic peak.

**Figure 6:**
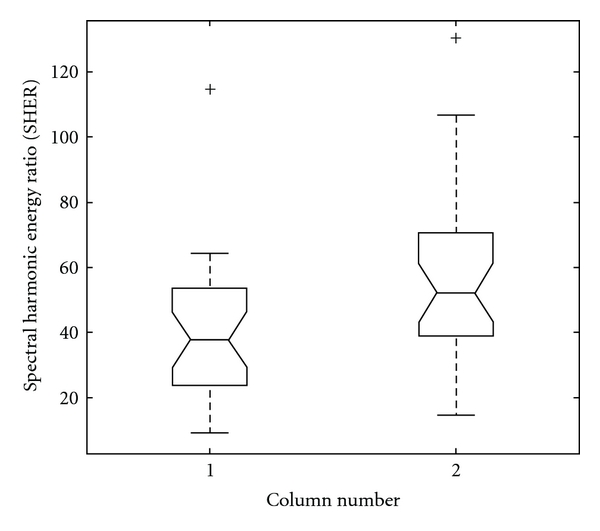
The box plot of the SHER of the N and P groups is demonstrated. Column 1 and 2, respectively, represent the N and P groups.

The measured arterial pulse at the left “Chun" for a typical normal subject in the N group and its corresponding power spectral density are shown in Figures 4(a) and 4(b), respectively. By observing Figure 4(b), we find that the harmonic peaks gradually decrease in order. The measured arterial pulse at the left “Chun" for a typical subject with palpitation in the P group and the related power spectral density are shown in Figures 5(a) and 5(b), respectively. From Figure 5(b), we perceive the abrupt decrease above the fourth harmonic. The SHER of this typical normal subject is 19.2 and that of the patient with palpitation is 76.2. The result reveals that the higher SHER value occurs in the patient subject due to the lesser energy in the fourth to sixth harmonic. Statistical results of SHER of the N and P groups are shown in Table 1 as mean ± SD and *P*-value. The mean SHER value of the P group (=57.7) is apparently larger than that of the N group (39.7). *P*-value (=.0066 <.01) exhibits the significantly higher SHER values of the P group than those of the N group. The box plot of SHER values of the N and P groups is illustrated in Figure 6. In other words, the statistical results of SHER verify our original inference that the harmonic energy above the fourth harmonic drops more significantly and more drastically in the patient group than in the normal group. The result reveals the clinical efficacy of SHER. 

## 4. Discussion

By the SHER index and the statistical method, we know that the spectral harmonics of the arterial pulse at the left “Chun" decrease progressively in normal subjects while they decrease abruptly in the fourth, fifth and sixth harmonics in patient subjects. This exhibits a close association between the patients with palpitation and the abrupt energy decrease of the arterial pulse in the fourth, fifth and sixth spectral harmonics. The reasons could be stated as follows. According to the organ resonance theory proposed by Wang et al. [13, 14] the higher the fundamental resonance frequency, the smaller the size of the resonance cavity. A smaller cavity has less blood volume, so less energy is required for the arterial pulse to push the blood into the smaller cavity. We thus know that the height of the harmonic peaks in the arterial pulse spectrum will gradually decrease under normal conditions as the harmonic frequency gradually increases; however, in contrast, an abrupt change in spectral harmonic distribution means disorder in the human body. From the viewpoint of the theory of TCM, the time-domain pulse will exhibit mild or relaxed strength for normal persons. Conversely, if stiffness, tightness, inflexibility or weakness occurs in the time-domain pulse, there will be some disorders in the human body [21]. In other words, the abrupt change of pulse strength, either increased or decreased, signifies health disorders. The change of pulse strength will lead to the variation of time-domain waveform, accounting for the variation in spectral distribution according to the theory of Fourier transform. Actually, “gradual decrease" implies balanced status; “abrupt decrease” implies unbalanced status in blood circulation and pulse diagnosis. “Unbalance" is an important factor causing disease in TCM [21–24]. Hence, the reason for the abrupt change in spectral distribution of the arterial pulse means that disorders can also be explained by the theory of TCM. SHER is defined as the energy ratio between low-order harmonics and high-order harmonics. If SHER is lower, the energy of high-order harmonics is higher. It means faster variations, that is, stiffness or tightness in time-domain waveform according to Fourier transform. Conversely, if SHER is higher, the energy of high-order harmonics is lower. It means slower variations, that is, weakness in time-domain waveform. Therefore, SHER is a simple and excellent index to reveal the balanced as well as the unbalanced state in blood circulation.

Furthermore, the constricted or blocked blood vessels, or diseased organs supplied by a group of arterioles or the thrombotic artery might cause blood redistribution in the arterial system, and thus lead to severe fluctuation or abrupt change in the pulse spectrum compared with the normal spectral distribution. In other words, there might be an increase or decrease in some specific frequency band of the arterial pulse. In the palpitation patients, decreases in the fourth, fifth and sixth harmonics are observed. In addition, both the small arteries and fast vibration of the artery wall are related to the high-order harmonics of the arterial pulse spectrum. We would also know that the abrupt decay in high-order harmonics (above the fourth) means the decreased ability of the heart to push the blood into small arteries or the decreased response of the artery wall to the fast vibration for the patients with palpitation.

Furthermore, recent studies have proved that the structural alterations in the microcirculation of low-resistance artery play an important role in organ damage. Remodeling (structural alterations) of the resistance vasculature plays a key role in the pathogenesis of essential hypertension, in which eutrophic remodeling allows the vessels to maintain an increased resistance without increased activation. Therefore, the tunica media to the internal lumen ratio of subcutaneous low-resistance arteries is increased and used as a superior predictor of cardiovascular events [25, 26]. The increased resistance of the low-resistance artery may lead to blood redistribution and cause cardiovascular-related diseases. The low-resistance artery corresponds to the small resonant cavity as well as to the high-order harmonic of the radial pulse spectrum. Hence, these researches provide possible reasons why there are some variations in this frequency band of high-order harmonics for cardiovascular-related patients.

Briefly, the redistribution of blood flowing into the organs has a strong relationship with physiological and pathological status. As a result, the spectral distribution of the arterial pulse holds much information about how the heart distributes the blood into the arterial system and to the organs. With regard to the time-domain waveform of the arterial pulse, there will be many different time-domain waveform patterns that correspond to different pulse spectral distributions through inverse Fourier transform. Now that the pulse spectral distribution has a relationship with physiological and pathological statuses, the time-domain waveform patterns are also related to the physiological and pathological statuses. That is why the doctor of TCM, using the pulsation of the radial artery at the wrist, can determine the physiological and pathological status of patients. These different time-domain pulse waveforms constitute the basis of the traditional 28 pulse patterns in the pulse diagnosis theory of TCM. The traditional pulse diagnosis technique of TCM has concentrated on the feeling of the time-domain pulse pattern for thousands of years. If we can extend the pulse diagnosis technique into the spectral domain, some features that are hard to find in the time domain or by the fingertips will be clearly displayed in the pulse spectral distribution. Through the use of both time-domain and spectral-domain analyses, we thus can have a more complete and comprehensive understanding of such a non-invasive pulse diagnosis technique. In conclusion, this analysis method, SHER, related to the spectral distribution of the arterial pulse, provides a novel viewpoint from which to observe the interaction between the heart and blood flowing into the organs or the arterial system. These concepts can be further applied to improve the clinical diagnosis not only in Western medicine but also in TCM.

## Supplementary Data

Supplementary Data are available at *eCAM* online.

## Funding

National Science Council of Taiwan under grant no: NSC 98-2221-E-324-043.

## Supplementary Material

The time-domain waveform of the instrumention without touching the hands is viewed as background noise.

## Figures and Tables

**Table 1:** The statistical results of SHER between N and P groups.

	SHER
N group	39.7 ± 20.9
P group	57.7 ± 27.9
*P*-value	.(0066 < .01)
