# Supplementary material for: Developing the Effective Method of Spectral Harmonic Energy Ratio to Analyze the Arterial Pulse Spectrum
Source: Evid Based Complement Alternat Med. 2011 Jan 11;2011:342462. doi: 10.1093/ecam/neq054 (PMC3152871; doi:10.1093/ecam/neq054)

Supplemental figure:

The time-domain waveform of the instrumention without touching the hands is viewed as background noise and shown in the following figure (a). Its power spectral density is shown in the following figure (b). The average power spectral density (0~10 Hz) is equal to $1.5 \times 10^{-15} V^2 / Hz$.

(a)

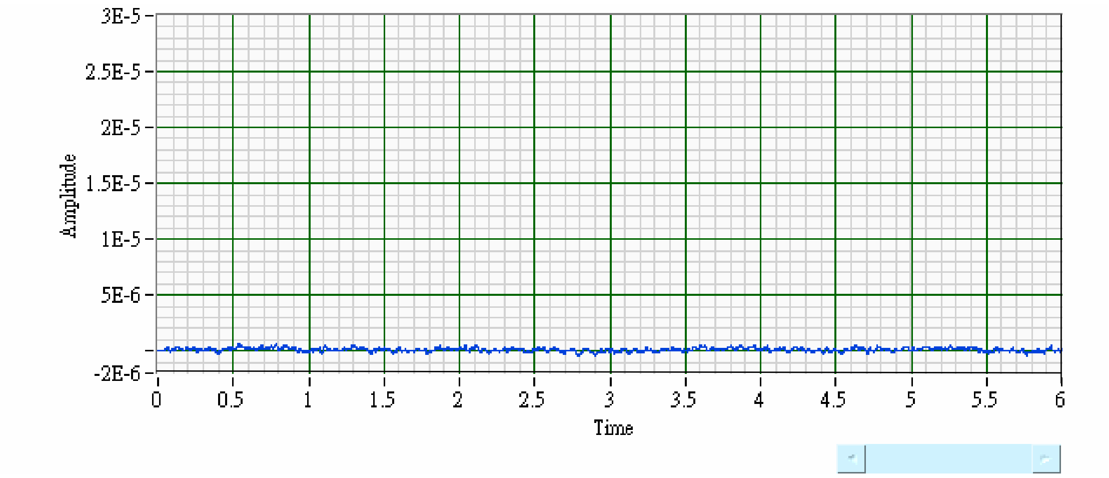

(b)

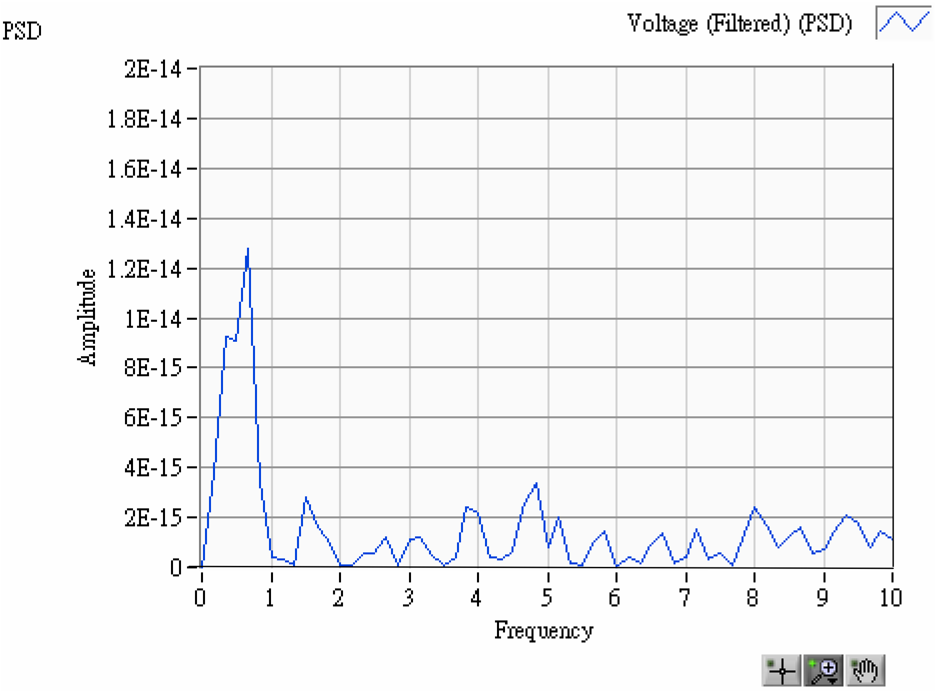

Supplement: Supplementary file 1 — The time-domain waveform of the instrumention without touching the hands is viewed as background noise. [file 342462.f1.pdf]
